# Assessing São Paulo’s public transport efficiency and coverage through data-driven modeling of autonomous vehicle and vehicle-as-a-service integration

**DOI:** 10.1038/s41598-025-32807-z

**Published:** 2025-12-23

**Authors:** Lucas Henrique L. Antonio, Sidney Junior C. Terenciani, Danilo M. Eler, Geraldo P. R. Filho, Lourenco A. Pereira Junior, Robson E. De Grande, Rodolfo I. Meneguette

**Affiliations:** 1 School of Sciences, São Paulo State University (UNESP), Av. Eng. Luís Edmundo Carrijo Coube, 17033-360 Bauru, São Paulo Brazil; 2 Faculty of Science and Technology, São Paulo State University (UNESP), R. Roberto Simonsen, 19060-900 Presidente Prudente, São Paulo Brazil; 3 Department of Exact and Technological Sciences, State University of Southwest Bahia (UESB), Estr. Bem Querer, Km-04, 45083-900 Vitória da Conquista, Bahia Brazil; 4 Aeronautics Institute of Technology (ITA), São José dos Campos, 12228-900 São Paulo Brazil; 5 Department of Computer Science, Brock University, 1812 Sir Isaac Brock Way, St. Catharines, Ontario Canada; 6 Institute of Mathematics and Computer Sciences (ICMC), University of São Paulo (USP), Av. Trabalhador São Carlense, 13566-590 São Carlos, São Paulo Brazil

**Keywords:** Energy and society, Engineering, Geography, Geography, Mathematics and computing, Science, technology and society, Social sciences

## Abstract

São Paulo, the largest Brazilian metropolis, faces complex challenges in urban mobility, exacerbated by growing population demands and the unequal distribution of public transport infrastructure. This study analyzed the efficiency of the city’s public transportation system using geospatial visualization techniques and data analysis. Significant disparities were identified between central areas, which exhibit higher density and connectivity, and peripheral neighbourhoods, which experience lower vehicle frequency and longer waiting times. To address these challenges, the study explored the potential of emerging technologies, such as autonomous vehicles and the Vehicle-as-a-Service (VaaS) model, to expand coverage and enhance public transportation efficiency. Autonomous vehicles can help reduce accidents caused by human error and alleviate traffic congestion, while the VaaS model offers an intelligent integration of vehicles and urban infrastructure, fostering a more flexible and scalable mobility system. Despite the promising prospects of these innovations, their implementation faces several challenges, including the need for specific regulations, robust technological infrastructure, and public acceptance. This study concludes that adopting analytical technologies and emerging solutions is crucial to transforming urban mobility in São Paulo, fostering a more efficient, inclusive, and sustainable public transportation system.

## Introduction

São Paulo – Brazil, one of the largest conurbations in the world, faces significant challenges in urban mobility. With a population nearing 12 million inhabitants, the city relies on a vast and diverse public transportation system to meet the daily commuting needs of its residents. Although São Paulo boasts an integrated network of subways, trains, and buses, buses remain the most utilized mode of transportation, particularly by residents of the city’s peripheral areas ^1^. It is estimated that the daily bus fleet comprises approximately 14,000 vehicles operating on more than 1,300 routes, covering nearly all regions of the city ^2^. The management of this complex system falls under the responsibility of São Paulo Transporte (SPTrans), which oversees routes, schedules, and the daily operation of public transportation in the city ^2^.

Despite its large fleet and vital role in São Paulo’s urban mobility, public transportation faces persistent challenges. Issues such as punctuality, route frequency, and service coverage are frequently criticized by users and experts alike ^1^, ^3^. These problems stem from overcrowding during peak hours, inadequate infrastructure in certain areas, and increasing passenger demand due to population growth and unplanned urban expansion ^1^, ^4^. The city’s historical development, characterized by a concentration of services and employment opportunities in central areas, has led to a growing demand for efficient public transport, particularly in peripheral zones where the system often struggles to meet the population’s needs ^4–6^.

Given these challenges, it is essential to explore innovative solutions that can enhance operational efficiency and expand coverage. Among the emerging technologies poised to transform the urban mobility landscape are autonomous vehicles (AVs) and the concept of Vehicle-as-a-Service (VaaS), which together have the potential to revolutionize how cities plan and provide public transportation.

However, despite the growing number of studies on smart mobility and autonomous transportation, there remains a research gap in quantitatively assessing how AV-based operations could improve the efficiency and spatial equity of existing public transport systems in large metropolitan areas, particularly in Latin America.

While previous research has explored autonomous mobility and public transport optimization separately, there is still a lack of integrated, data-driven studies connecting real data with predictive modeling of AV and VaaS scenarios. This methodological gap represents a key motivation for the present research.

Despite the recent surge in interest around smart mobility, studies that quantitatively integrate real-world General Transit Feed Specification (GTFS) data with predictive modeling and autonomous vehicles (AVs) or the Vehicle-as-a-Service (VaaS) model remain comparatively scarce. A growing body of research has explored the analytical potential of GTFS data for understanding public transport operations and spatial accessibility. These studies represent important methodological progress, yet most remain focused on visualization or diagnostic applications, rather than predictive or integrative modeling capable of supporting operational planning.

There are several works that handled GTFS data for visualization, performance, and analysis. Prommaharaj et al. (2020) developed a GTFS-based visualization tool to display spatiotemporal patterns of transit services, illustrating the potential of GTFS for diagnostic analysis ^7^. Fortin et al. (2021) proposed a systematic framework for transit network evaluation grounded in GTFS indicators, demonstrating how standardized data can support performance benchmarking ^8^. More recently, Liu et al. (2025) introduced GTFS2STN, an advanced tool that converts GTFS data into spatiotemporal networks, enabling in-depth analysis of travel time variability and accessibility dynamics across complex transit systems ^9^. However, these works do not propose a practical analysis of the impact of autonomous car mobility and perform an analysis of predictive models on the study.

Beyond these preliminary efforts, recent research has increasingly evolved toward data-driven and simulation-oriented frameworks that integrate GTFS datasets with advanced predictive and optimization techniques. Ferreira et al. (2025) introduced the Salvador Urban Network Transportation (SUNT) dataset, offering a comprehensive GTFS-derived structure to evaluate the operational efficiency of large-scale transit systems in Brazil^10^. Ribeiro et al. (2025) employed GTFS and ticketing data within a digital twin environment to model multimodal service performance under varying demand conditions^11^. In parallel, Tóth et al. (2025) developed interactive visualization methods for GTFS and GTFS-RT feeds to identify spatial and temporal patterns of service delays in Budapest^12^. Similarly, Perez et al. (2024), Sargiotis (2025), and Vu et al. (2025) proposed AI-driven and agent-based transport models that leverage GTFS data to simulate adaptive and sustainable mobility scenarios^13–15^. Complementarily, Colella et al. (2025) applied data-driven modeling techniques to tram systems, emphasizing the role of predictive analytics in advancing energy-efficient and intelligent urban transport operations^16^.

Despite these methodological advancements, empirical applications combining GTFS data with predictive modeling remain scarce, particularly those exploring the transformative potential of Connected and Autonomous Vehicles (CAVs) and Vehicle-as-a-Service (VaaS) paradigms in large metropolitan contexts. The present study addresses this research gap by developing a regression-based analytical framework grounded in São Paulo’s public transit data, thereby offering a reproducible and data-centric methodology for assessing operational efficiency and projecting the implications of future automation and service integration.

Building upon these methodological advances, the present study integrates empirical operational datasets, predictive modeling, and AV/VaaS scenario simulations to deliver a comprehensive and quantitative evaluation of public transport performance. This integrative approach combines methodological rigor with practical applicability, moving beyond descriptive or conceptual analyses toward a reproducible, data-driven framework suitable for metropolitan-scale studies. The originality of this research lies precisely in this convergence: the fusion of real-world GTFS operational data with predictive modeling and AV-based simulations enables a spatially explicit and empirically grounded assessment of public transportation systems. By bridging empirical evidence and technological foresight, the study advances the methodological frontier of urban mobility analysis.

This study’s originality lies in its integration of real-world GTFS operational data with predictive modeling and AV-based simulation scenarios, providing a quantitative and spatially explicit assessment of public transport performance. By combining these methods, the study advances beyond conceptual frameworks to offer a reproducible, data-driven approach.

Building on this methodological foundation, it is also essential to outline the technological principles that underpin these innovations in mobility. Autonomous vehicles, equipped with sensors, cameras, artificial intelligence, and data-processing systems, can operate without human intervention, helping to reduce operational inefficiencies and improve system performance. Although still in the testing phase globally, this technology promises significant improvements in urban mobility. The VaaS model expands on this concept by integrating autonomous vehicles into intelligent, connected platforms that provide infrastructure-level services to cities. In this model, vehicles not only transport passengers but also perform distributed functions such as data collection, routing coordination, and communication with urban infrastructure, contributing to more adaptive and responsive transport operations ^17,18^.

Understanding the impact of autonomous vehicles and the VaaS model in São Paulo is essential for evaluating their potential benefits to urban mobility. Given the city’s complex urban network and mobility challenges, São Paulo serves as an ideal case for exploring these innovations. By analyzing public transportation data, patterns, and opportunities for improvement can be identified, informing strategic decisions to maximize the benefits of these technologies. This research investigates how the integration of autonomous vehicles (AVs) within the Vehicle-as-a-Service (VaaS) model can enhance the efficiency, equity, and spatial coverage of São Paulo’s public transport system. Using General Transit Feed Specification (GTFS) datasets and predictive modeling, the study quantifies the potential improvements in travel times and service distribution that AV-based operations could introduce. Thus, the main contributions of this work are as follows:

- Comprehensive analysis of São Paulo’s public transportation system, integrating GTFS datasets and public security data to identify key inefficiencies;
- Evaluation of the potential impact of autonomous vehicles (AVs) and the Vehicle-as-a-Service (VaaS) model on improving transit efficiency, coverage, and accessibility;
- Implementation of advanced visualization techniques, such as heatmaps and interactive maps, to highlight underserved areas and assess the impact of AVs on reducing wait times; and
- Provision of actionable insights to guide urban mobility strategies and policy-making.
- Evaluation of three different regression models for Prediction.

The remainder of this article is organized as follows. Section presents the key theoretical frameworks. Section details the proposed methodology, while Section outlines the main techniques employed in this study, addressing the questions raised. Section introduces a statistical analysis of spatial and operational patterns, focusing on the relationship between proximity to transit hubs and service demand. Section highlights the primary results obtained and provides a discussion of these findings. Finally, Section presents the concluding remarks and future challenges.

## Theoretical framework

The theoretical foundation of this study establishes the key concepts and theoretical pillars necessary to understand the topic under analysis. This section explores the main concepts related to autonomous vehicle technologies and the Vehicle-as-a-Service (VaaS) paradigm, situating them within the context of smart cities. The objective is to provide a solid basis for understanding how these technologies are structured and how they can be integrated into more efficient and sustainable urban systems.

Initially, the principles governing the operation of autonomous vehicles are discussed, highlighting the technological advancements that enable their operation without human intervention. Next, the concept of VaaS is presented, which transforms vehicles into active elements of urban infrastructures, performing functions beyond transportation.

Furthermore, the theoretical foundation addresses the impact of these technologies within the context of smart cities, emphasizing their contribution to improving urban mobility and resource management. The challenges associated with the implementation of these innovations are also explored.

By integrating these concepts and discussing their interrelations, the theoretical foundation provides a comprehensive overview that supports the analysis proposed in this study, offering the necessary basis to understand the potential and limitations of the topic under consideration.

### Autonomous cars and vehicles-as-a-service

Autonomous cars operate without human intervention, using advanced technologies like sensors, cameras, AI, and data processing. Systems such as LIDAR and GPS help the vehicle detect obstacles, other vehicles, pedestrians, and traffic signs, enabling real-time decision-making for safe and efficient driving ^19^. The development of this technology gained traction in the early 2000s, with projects like Google’s Waymo, and has since seen heavy investments aimed at achieving Level 5 autonomy, where vehicles are fully independent ^20,21^.

Vehicle-as-a-Service (VaaS) transforms vehicles from simple transportation tools into integral elements of smart city infrastructure. In this model, connected and autonomous vehicles are part of distributed urban systems, performing functions like data processing, storage, and real-time communication. Supported by technologies like fog computing, VaaS enables scalable networks that enhance the urban ecosystem ^17,19,22,23^. VaaS applications in smart cities include collaborating with traffic control devices, monitoring traffic, and supporting functions such as emergency management and environmental monitoring, positioning vehicles as interconnected elements of a sustainable urban network ^19,23^.

### Impact on smart cities

As the development of autonomous cars progresses, several cities worldwide have begun testing these technologies in public transportation, yielding promising results, though challenges remain. One of the most well-known examples is the test conducted in Phoenix, Arizona, by Waymo’s autonomous vehicles. Since 2017, Waymo has been operating an autonomous transportation service in specific areas of the city. This initiative allowed passengers to travel in driverless vehicles along pre-defined routes, helping to reduce congestion and provide greater reliability in transportation, especially during peak hours ^24^.

In Europe, Helsinki, Finland, stands out as one of the leading cities in smart mobility initiatives, particularly through the use of autonomous vehicles. One of the most notable projects is the Smart Mobility initiative, led by Forum Virium, which encompasses multiple research fronts ^25^. Key themes include the use of low-carbon energy, the development of advanced vehicles, and the creation of intelligent mobility and transportation systems. Part of these initiatives involves testing electric autonomous buses, which are being explored as a solution for short-distance transportation ^25,26^.

Additionally, Helsinki has been exploring ways to integrate these vehicles into broader urban mobility systems as part of projects focused on urban logistics and intelligent passenger flow management. For instance, at ferry terminals connecting Helsinki to Tallinn, solutions are being tested that combine automated queue management systems to reduce congestion and smart ticketing services to streamline passenger boarding ^26^. These initiatives demonstrate how autonomous vehicles can be integrated into a larger smart transportation ecosystem, not only improving the efficiency of public transit but also reducing environmental impact ^26^.

In Asia, cities like Singapore are at the forefront of testing autonomous vehicles within public transportation networks. In Singapore, the Land Transport Authority has been collaborating with technology companies to develop and test autonomous vehicles operating in specific zones of the city ^27,28^. Beyond reducing congestion, these tests have demonstrated how integration with smart infrastructures, such as traffic lights and traffic sensors, can enhance overall urban transportation efficiency by enabling more dynamic and adaptive routes to meet passengers’ real-time needs ^27,28^.

Although the impact of autonomous vehicles on public transportation is largely positive, promising improvements in punctuality, reduced congestion, and expanded coverage in underserved areas, challenges persist. Adapting urban infrastructure to support this technology represents a significant obstacle, particularly in ensuring constant communication between vehicles and traffic systems. In areas with limited wireless network transmission capacity, autonomous vehicles face difficulties in staying updated on the position and routes of other vehicles, which is crucial for planning paths and avoiding collisions ^29^. However, in regions with robust network infrastructure, vehicles could share real-time information about their routes and movements, enhancing both safety and efficiency.

Furthermore, many cities lack roads and traffic systems suitable for autonomous vehicles. Additionally, the cost of implementation remains a significant barrier, particularly for public transportation systems that are already facing financial challenges ^30^.

## Methodology

The methodology employed in this study analyzed São Paulo’s public transportation system using data provided by SPTrans in GTFS format. Covering the period from October to November, the dataset detailed routes, stops, schedules, frequencies, and geographical layouts, offering a robust foundation for examining efficiency and service coverage.

To address challenges such as inconsistencies and high data volume, a preprocessing stage was implemented to consolidate daily GTFS files into unified datasets, ensuring consistency and completeness. Sampling techniques were subsequently applied to optimize computational efficiency while preserving key operational patterns such as stop recurrence and frequency variations.

The study focused on four key research questions: identifying routes with the longest and shortest wait times, and assessing areas with limited public transportation coverage. Data visualization techniques, including heatmaps and interactive maps, played a central role in identifying patterns and interpreting findings.

1. Which bus routes demonstrate the greatest inefficiencies in terms of waiting times and service regularity within São Paulo’s public transport system?
2. How could the adoption of autonomous vehicle (AV) operations contribute to reducing travel times and optimizing service frequency across existing routes?
3. Which regions of São Paulo remain underserved by public transport, and how do current coverage patterns reveal spatial inequities in service distribution?
4. How could the integration of AV and Vehicle-as-a-Service (VaaS) models enhance coverage and service equity in underserved areas of São Paulo?

To answer these questions, various data visualization and analysis techniques were applied to assist in identifying patterns and interpreting relevant information about the public transportation system. These visualizations were selected according to the specific needs of each question and will be detailed in the Results section, where the insights gained from each analysis are presented and discussed.

**Fig. 1:**
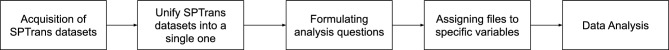
Key steps of the methodology proposed.

Figure 1 illustrates the key steps of the proposed methodology. The process begins with data acquisition, in which datasets from SPTrans are collected. Next, the unification step consolidates daily GTFS files into a single, cohesive dataset to ensure consistency and completeness. The subsequent stage involves formulating research questions that define the primary focus of the study, such as identifying wait times and evaluating operational efficiency. Afterward, data organization assigns files to specific variables, structuring the datasets into manageable components for analysis–such as trips, stops, and frequencies. Finally, the data analysis phase employs visualization and computational techniques to address the research questions, generating insights into the efficiency and coverage of the public transportation system.

### Dataset and preprocessing

This research utilized data provided by SPTrans through the GTFS (General Transit Feed Specification), a standard format for the exchange of static public transportation information. The dataset was obtained from the developer section of the SPTrans portal and covers the period from October to November.

The GTFS dataset includes a series of essential files that provide a comprehensive view of the operation of public transportation in São Paulo. These files are organized into modules, each containing specific information that, together, detail aspects such as routes, stops, schedules, and frequencies of bus lines. Below are descriptions of some of the main files and their respective roles in the analysis:

- *Fare_rules* and *Fare_attributes*: Contain information about fares, rules, and fare structures.
- *Calendar*: Specifies the days and times each route operates.
- *Agency*: Provides details about the agency responsible for transportation, in this case, SPTrans, including relevant administrative data.
- *Frequencies*: Records the frequency of buses on each route throughout the day.
- *Routes* and *Trips*: Specify the scheduled routes and trips, including the relationship between each route and its stops.
- *Stop_times*: Indicates the arrival and departure times of buses at each stop.
- *Stops*: Lists the locations and identifiers of each stop.
- *Shapes*: Describes the exact layout of the routes.

These variables form a comprehensive foundation for exploring essential aspects of public transportation operations, such as punctuality, route frequency, geographical coverage, and travelled paths. However, working with this data presents some challenges. Since the GTFS is updated daily, it is common for small registration discrepancies to occur, including missing or inconsistent data on specific days. Such issues can significantly impact the analysis, requiring preprocessing to ensure that all records are consistent and complete throughout the selected period.

The extensive volume of data poses a significant challenge, with millions of entries generated monthly, requiring robust computational resources for analysis. To ensure the integrity of analyses focused on stops and wait times, a specialized treatment process was adopted. Instead of merely removing duplicate records, all datasets were consolidated into a comprehensive database.

For specific analyses, the database was adjusted by reducing the sample size in controlled proportions (e.g., 0.01 or 0.001), balancing computational efficiency with the preservation of critical patterns such as stop recurrence and frequency variations. This approach enabled in-depth insights while optimizing computational requirements for each study phase.

### Development environment and libraries used

Data analysis and manipulation were conducted using Google Colab integrated with Google Drive, ensuring seamless access to files in designated directories.

Key libraries like Pandas, NumPy, Matplotlib, Seaborn, Folium, and OS were employed for their efficiency. Pandas structured the data in dataframes, while NumPy facilitated numerical operations and vectorized calculations. For visualization, Matplotlib and Seaborn produced scatter plots, box plots, and heatmaps, revealing data patterns. Folium enabled interactive maps for geographical analysis of routes and stops, and the OS library managed directories and file verification, streamlining processing and automating workflows.

### Dataset consolidation

To support consistent monthly analysis, SPTrans’s daily GTFS data was consolidated into unified datasets for categories such as frequencies, trips, and stops, ensuring data integrity and simplifying processing.

The consolidation involved two key stages. First, a structured organization of daily files was established, including folder arrangements and a logical framework for automated data reading. Next, a function systematically processed these folders, reading daily files, temporarily storing the data, and monitoring for missing files or inconsistencies. Alerts were generated for any missing files, enabling prompt corrections. Finally, the daily data was concatenated into consolidated datasets for each category, centralizing the information and facilitating comprehensive analysis across the period.

The consolidated dataset formed the foundation for subsequent analyses, offering a robust and organized structure for all stages of the study.

Each file was imported and assigned to specific variables, such as frequencies, trips, routes, stop_times, and stops, which represented key data categories for addressing the research questions. This step ensured systematic organization and maintained data integrity, enabling a detailed exploration of public transportation aspects, including travel regularity, stop mapping, and service intervals. This preparatory phase provided a solid basis for the in-depth analyses and visualizations required to meet the research objectives effectively.

## Processing data

This study explores the operational and spatial dynamics of São Paulo’s public transport system rather than focusing on the graphical design or coding details.

### Bus route wait time analysis

The first stage of visualization focused on identifying operational inefficiencies by analyzing bus route waiting times. Using the headway_secs variable, representing the interval between vehicles on the same route, average waiting times were calculated for each bus line and aggregated to capture network-wide variability.

Descriptive plots were then generated to highlight the distribution of waiting times and identify the routes with the longest delays. The analysis also compared waiting times to the number of trips performed on each route, revealing relationships between service frequency and demand levels throughout the day.

To capture temporal patterns, four operational intervals were used – morning peak (06:00–09:00), midday (09:00–12:00), afternoon (12:00–15:00), and evening peak (15:00–18:00). Heatmaps were employed to visualize variations across these intervals, allowing the identification of mismatches between demand concentration and service regularity.

### Urban gaps in transit service

The second stage examined spatial disparities in public transport coverage. Geospatial analysis was conducted to map bus stop density, vehicle frequency, and trip recurrence across São Paulo’s districts. Aggregation and segmentation techniques were applied to identify areas with lower accessibility or service concentration.

Heatmaps and scatter plots provided a spatial overview of service distribution, distinguishing high-demand zones from underserved regions. Kernel Density Estimation (KDE) was used to generate a continuous representation of bus stop density, enabling the identification of clusters and low-density areas without relying solely on discrete points.

Terminals and subway stations were also mapped to contextualize accessibility and multimodal integration within the city’s network. The combination of these spatial visualizations offered a comprehensive perspective on service equity, highlighting the regions most in need of infrastructure or operational improvement.

### Autonomous vehicles and service expansion

The introduction of autonomous vehicles into public transportation systems represents an innovation with significant potential to improve coverage and accessibility, particularly in areas currently receiving limited or irregular service. These underserved regions often face specific challenges, such as low population density, limited road infrastructure, and fluctuating transportation demand throughout the day.

By integrating artificial intelligence, advanced sensing technologies, and driverless operation, autonomous vehicles offer unique features that distinguish them from conventional modes of transportation. These capabilities enable more efficient and adaptable service. Below, the key ways autonomous vehicles could benefit such areas are explored, providing a theoretical perspective on how this technology could reshape urban mobility.

#### Service enhancement via autonomous vehicles

The implementation of autonomous vehicles offers an opportunity to enhance public transportation coverage while significantly improving traffic flow and overall mobility. Operating under the Vehicle as a Service (VaaS) model, autonomous vehicles can function as interconnected “assistants,” sharing data and resources to enhance traffic safety and efficiency. With precise scheduling and integrated networks, these vehicles are capable of reducing congestion by preventing overloading on specific routes and times. Additionally, through real-time data collection and sharing, autonomous vehicles can identify road hazards, adjust traffic flow intelligently, and minimize bottlenecks, an advantage particularly valuable in peripheral urban areas where traffic is often impacted by private vehicle movement ^17,22,23,25^.

Reduced congestion makes autonomous public transport a more attractive option, encouraging the use of collective modes of transportation while discouraging reliance on private vehicles, which often exacerbate traffic in underserved areas ^31,32^. Improved traffic flow leads to faster and more predictable travel times, directly benefiting transportation coverage by enabling autonomous vehicles to complete more trips in less time, serving a broader range of areas with greater regularity.

Another potential enhancement involves integrating traffic sensors, cameras, and communication networks to monitor vehicle flow and automatically adjust traffic light synchronization, optimizing transit in high-density regions and enabling more efficient transportation. Introducing a dynamic electronic tolling system that adjusts fees based on congestion could further encourage alternative routes, alleviating pressure on critical areas. Additionally, contactless ticketing systems and autonomous vehicles adapted to serve the elderly and individuals with disabilities would expand accessibility and promote inclusivity in public transit. Inspired by Singapore’s model, these features contribute to a sustainable, “car-lite” mobility system that prioritizes public transportation and extends coverage to currently underserved areas ^27,28,31–33^.

By increasing frequency and improving service quality, autonomous vehicles have the potential to transform public transportation’s reach, effectively connecting less-served regions and fostering a more efficient and sustainable mobility system across both urban and peripheral areas.

#### Reduction of operational costs

Level 5 autonomous vehicles, operating without human intervention, significantly reduce public transportation costs, particularly in underserved regions with limited demand. By eliminating driver salaries and enabling extended service hours, autonomous buses can expand coverage and provide continuous service. Trials in cities such as Austin and Arlington have shown operational savings and efficiency improvements through route optimization ^20,34–37^.

Fully electric autonomous vehicles further lower costs by eliminating fossil fuel reliance, making operations economically sustainable, even in low-return areas. This enables the integration of remote regions into public transit networks without financial strain, promoting a more inclusive and accessible transportation system ^37–40^.

#### Reaching infrastructure-limited zones

The introduction of autonomous vehicle technology has the potential to significantly enhance public transportation by expanding its reach to underserved and hard-to-reach areas. In regions with low population density or limited infrastructure, where conventional buses are impractical due to high operational costs, smaller autonomous vehicles can offer more flexible and frequent services, providing better coverage and accessibility. This flexibility allows for tailored, on-demand routes that can better serve remote neighbourhoods and streets, which would otherwise remain disconnected from the public transport system. As a result, autonomous vehicles provide an efficient and scalable solution to meet the diverse transportation needs of underserved communities, helping to improve overall mobility and inclusion in urban and peripheral areas ^41–45^.

### Predictive mobility for segment time

A supervised regression model was designed and implemented, aiming at predicting the duration of individual transit segments within São Paulo’s public transportation system. The target variable represents the elapsed time in seconds between a vehicle’s departure from one stop and its arrival at the next, calculated from the GTFS feed’s departure_time and arrival_time fields. The objective was to model temporal and spatial patterns that affect route timing and network efficiency.

The initial preprocessing phase involved converting GTFS time strings into Timedelta objects to enable arithmetic operations. Negative or zero durations were excluded, as they represent inconsistencies in the data. Only valid segments with positive travel times were retained. To enhance the model’s ability to capture behavioural trends, additional features were engineered from the timestamp data. These included the hour of departure and the day of the week, allowing the model to incorporate temporal patterns related to traffic and operational schedules. Categorical variables, such as route identifiers, were encoded numerically to ensure compatibility with the regression algorithms. The final feature set included variables related to trip direction, stop sequence, geographic coordinates of the stops, and temporal attributes. The modelling dataset was then divided into training and testing subsets using an 80/20 split with a fixed random seed to ensure reproducibility.

To account for the varying sensitivity of different algorithms to feature scale, standardized versions of the predictor variables were generated using z-score normalization via StandardScaler. This transformation was applied exclusively to the training data and then used to transform the testing set, avoiding data leakage. Algorithms such as k-Nearest Neighbours (KNN), which are sensitive to feature magnitude, were trained on these standardized features, while tree-based models operated on the original scale.

Three regression algorithms were tested and optimized through grid search with 3-fold cross-validation, using negative mean squared error as the scoring metric. Decision Tree Regressor models were tuned using a range of hyperparameters related to maximum depth and minimum sample thresholds. These models offered fast computation and interpretability but were sensitive to overfitting on complex patterns. Random Forest Regressors were then applied to the same data, introducing ensemble learning and greater robustness. Variations in the number of estimators, depth, and split thresholds were tested to find the optimal configuration. Finally, KNN regressors were trained on the scaled dataset, evaluating different numbers of neighbours and distance-weighting strategies. While KNN provided a non-parametric alternative, its predictive power was generally lower compared to the tree-based approaches.

Across all models, performance was assessed using both Root Mean Square Error (RMSE) and the coefficient of determination (R²) on the test dataset. Among the three models, the Random Forest Regressor achieved the best balance between predictive accuracy and computational efficiency, exhibiting superior generalization capabilities. The model demonstrated an ability to capture nonlinear patterns in travel times, making it a promising tool for integration into operational planning and service reliability assessments.

#### Autonomous vehicles on travel times

To investigate the potential impact of autonomous vehicles on travel times within the São Paulo public transit system, we developed a simulation methodology that builds upon the previously trained predictive model. The aim was to model how systematic improvements brought by autonomous fleets, such as real-time traffic responsiveness, smoother navigation, and route optimization, could translate into reductions in segment-level travel duration. Two complementary code blocks were developed to address this problem, each with a specific analytical focus but based on the same simulation architecture.

The first simulation script centred on modifying predicted travel times generated by the previously optimized Random Forest model. After retraining the model on the original dataset with the best hyperparameter configuration (125 estimators, maximum depth of 20, minimum of 10 samples to split, and 2 samples per leaf), the model was used to predict segment durations on the test set. These predictions were then used as a proxy for the expected travel time under current operating conditions. Two hypothetical scenarios were simulated from these baseline predictions. In the first scenario, a uniform 15% reduction in travel time was applied across all predicted values, simulating a fleet-wide improvement due to continuous operational optimization. In the second scenario, a tiered approach was used: for segments with predicted durations above the 75th percentile, a 30% reduction was applied, reflecting enhanced performance in highly congested or delayed areas, while the remaining segments retained the 15% baseline reduction. The result of this first script was a set of adjusted predicted durations under both scenarios, used to compare the baseline with potential autonomous system behaviour.

The second simulation script employed a slightly different logic to assess these same scenarios but with a focus on time savings estimation rather than regression error metrics. Rather than comparing predicted times to observed durations, the emphasis was on calculating the expected reduction in travel time per segment. In this approach, the model’s predictions under current conditions were treated as the base scenario. The 15% and 30% reduction factors were then applied following the same logic as in the first script, but the adjusted values were directly compared to the original predictions themselves. This enabled a quantification of average time savings per segment for each scenario. Importantly, these simulations served as analytical tools for exploring operational impacts and did not aim to improve model accuracy or alter the learning pipeline.

Both simulation scripts are complementary; the first provides the predicted duration profiles under different conditions, while the second estimates how much time could realistically be saved with autonomous fleet deployment. Together, these analyses establish the foundation for the subsequent visual evaluation of results.

To support the simulated scenarios of autonomous fleet deployment, two graphical outputs were created to provide visual insights into the potential improvements in the travel segment durations. These visualizations serve as analytical summaries, helping communicate how the two proposed scenarios, uniform optimization and targeted congestion mitigation, differ in impact and operational behaviour.

The first graph was designed to illustrate the average time savings per segment, comparing the two scenarios against the baseline case. After generating adjusted predictions under both the uniform 15% reduction and the conditional 30% reduction for slower segments, the mean improvement was calculated as the difference between the original predictions and each adjusted scenario. These values were then visualized using a comparative bar chart. Each bar corresponds to a scenario and represents the average time saved in minutes, allowing for a direct and intuitive comparison of operational gains. This graph effectively highlights the relative efficiency gains introduced by autonomous routing and adaptive congestion handling. The second graph focused on comparing the absolute predicted travel durations across all three scenarios: current system behaviour, autonomous fleet with uniform optimization, and autonomous fleet with targeted mitigation of high-delay segments. The mean predicted duration for each case was calculated and presented in a bar chart, using a reversed orange-red palette to visually encode the shift from longer to shorter durations. In addition to displaying the raw average durations in minutes, this chart also annotates the relative percentage reduction for the autonomous scenarios, thereby offering a dual-layer perspective: absolute durations and proportional improvement. By placing all scenarios side by side, this visualization enables readers to understand both the direct and relative impacts of each simulated strategy. Together, these two figures form a coherent visualization framework that reinforces the methodological rigour of the simulation process.

## Analysis and results

This study integrates quantitative metrics to investigate the spatial and operational dynamics of São Paulo’s public transport network. By applying statistical techniques to geospatial and service data, we aim to uncover structured relationships that govern the distribution and intensity of transit activity. The inclusion of these metrics allows for a more systematic understanding of how proximity, frequency, and spatial configuration may influence service patterns within the network.

To quantitatively assess how proximity to metro terminals might influence transit usage, we began by measuring the linear relationship between the distance from each stop to the nearest terminal and the corresponding trip volume. Applying the Pearson correlation test revealed a statistically significant negative relationship, though of very weak magnitude:

$$\begin{aligned} r \approx -0.113, \quad p < 0.001 \end{aligned}$$

This result indicates that, based on the analyzed data, there’s only a minimal tendency for stops located closer to high-capacity metro infrastructure to support a slightly greater volume of trips. However, proximity to metro terminals, by itself, proves to be a minor influencing factor on the number of trips. This suggests that other elements, such as surrounding population density, the availability of commerce and services, or integration with other public and private transport modalities, likely exert a considerably greater impact on passenger flow

To further dissect the relationship between proximity to metro terminals and trip volume, stops were categorized into “near” and “far” groups based on the median distance to the high-capacity infrastructure. A Welch’s t-test for independent samples was then conducted to compare the mean trip volume between these two groups. This analysis yielded a T-statistic of 5.565 and a P-value of 2.708e-08. With mean trip volumes of 2114.91 for the “near” group and 1835.49 for the “far” group, these results indicated a statistically significant difference. The notably higher average trip volume in stops closer to terminals suggests that proximity to these key transit hubs plays a meaningful role in attracting a greater number of passengers, directly impacting the operational efficiency and ridership patterns within the network. This finding reinforces the intuitive understanding that ease of access to major transport nodes is a significant driver of public transport usage.

### Results

The results of the analysis will be presented according to the four research questions previously defined in the methodology. Each following section will detail the findings corresponding to each question, providing a comprehensive and detailed view of the observations made.

### Bus route wait time analysis

#### Average wait times

**Fig. 2:**
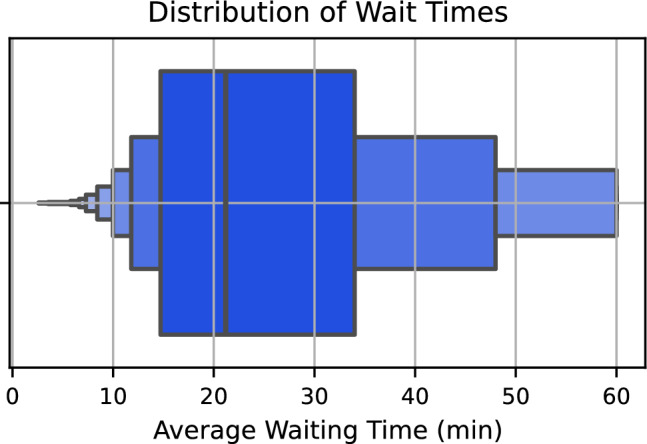
Average wait times by route.

The analysis of wait times (Fig. 2), we can observe that the average wait time generally ranges from 25 to 30 minutes, representing the highest concentration of data.

This result aligns with the research by Rede Nossa São Paulo, “Viver em SP 2024: Mobilidade Urbana,” which reports an average wait time of 24 minutes for buses at stops and terminals. However, the box plot includes data from buses, trains, and subways, providing a broader analysis of the public transportation system. Thus, while the research focuses on buses, the box plot offers a more comprehensive view of wait times across all modes of transport.

In addition, the image highlights the presence of significant outliers, especially with wait times of up to 60 minutes. The high number of these outliers is concerning and may indicate serious issues or considerable variability in the waiting system. The observation of these outliers provided valuable insights that guided the next phase of the analysis.

#### Demand for trips vs. wait time

To complement the investigation of the first research question, this analysis was conducted to provide a comprehensive and detailed view of travel demand and average wait times throughout the day.

**Fig. 3:**
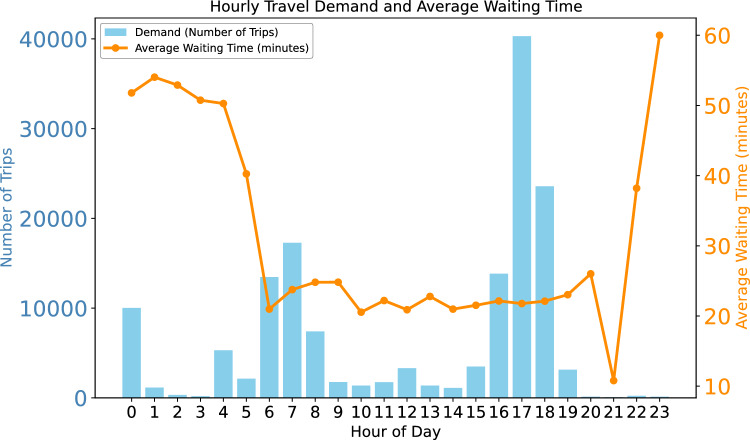
Describe the relation between travel demand and average waiting times across four operational time-of-day intervals.

Figure 3 summarizes the relationship between travel demand and average waiting times across four operational time-of-day intervals. Peak demand is concentrated during the morning peak (06:00–09:00) and evening peak (16:00–19:00), corresponding to typical commuting periods in São Paulo. Higher vehicle frequencies result in shorter average wait times during these intervals, reflecting an adaptive operational response to increased passenger flow. Conversely, off-peak periods such as the early morning (00:00–04:00) and late evening (21:00–23:00) show the longest average wait times, consistent with reduced service frequency and lower passenger demand.

### Urban gaps in transit service

**Fig. 4:**
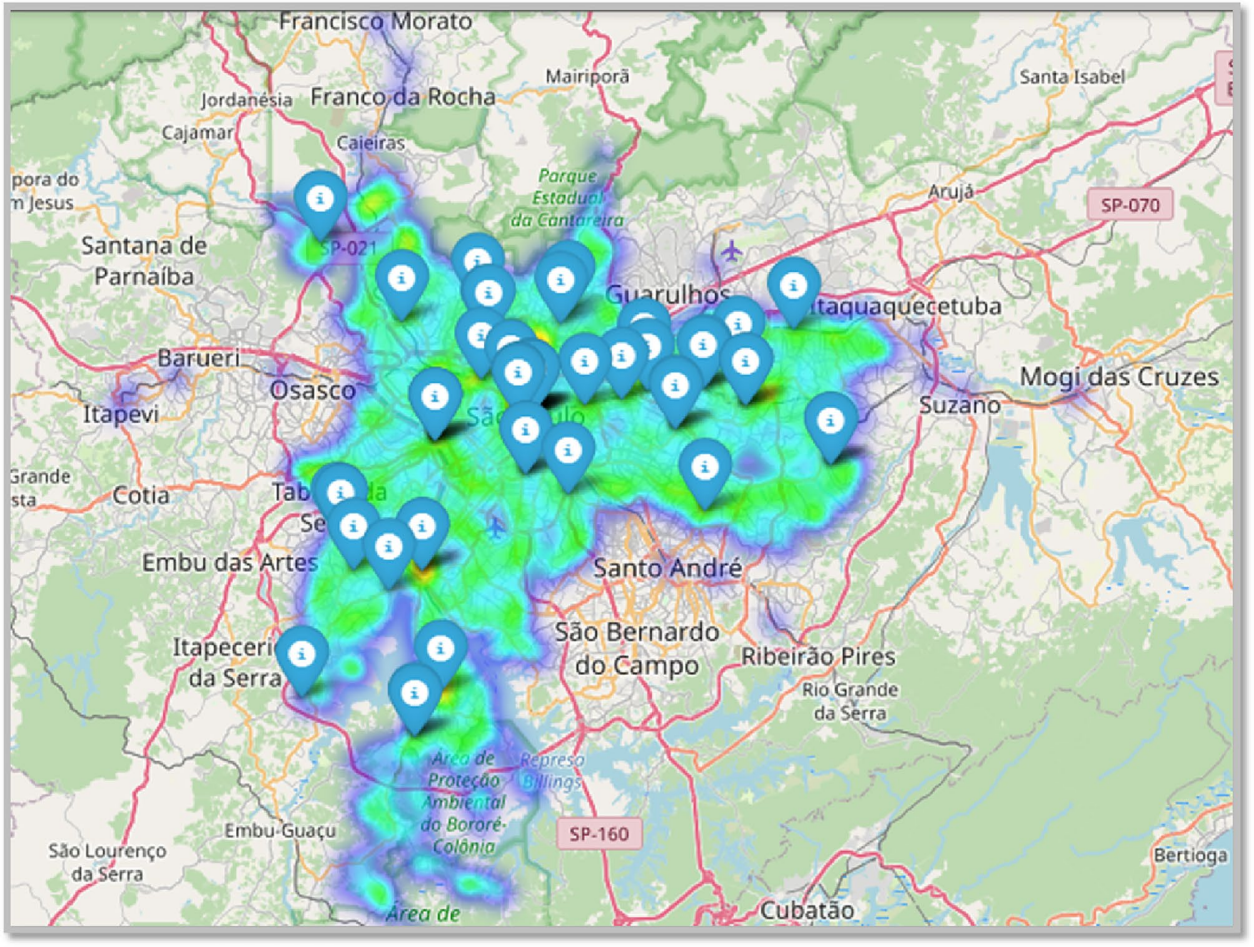
Geospatial visualization with heatmap and locations of public transport terminals and metro stations in São Paulo. Generated using the Folium library version 0.20.0 (https://github.com/python-visualization/folium) with OpenStreetMap (https://www.openstreetmap.org/#map=14/-23.55565/-46.63233) as the tile provider within the Folium function.

From the visualization presented on the map in Fig. 4, clear patterns of public transportation distribution and coverage in São Paulo can be observed. The analysis reveals that the highest concentration of public transport points is located in the central region of the city, depicted in colours, such as yellow and green. These central areas, characterized by higher population density and economic activity, have a more robust transportation infrastructure, offering better accessibility and coverage for users.

Conversely, the peripheral areas of the city, represented in blue, indicate lower coverage of the public transportation system. These regions, farther from the center, have fewer access points to public transport, which can lead to greater mobility challenges for residents. The lower concentration of transport points in these areas suggests a lack of infrastructure.

The relationship between the proximity of transport points and coverage quality is evident: the closer to the city center, the better the coverage and access frequency to public transport, whereas in peripheral areas, the coverage tends to be sparser.

#### Trip frequency and the location of terminals and stations

**Fig. 5:**
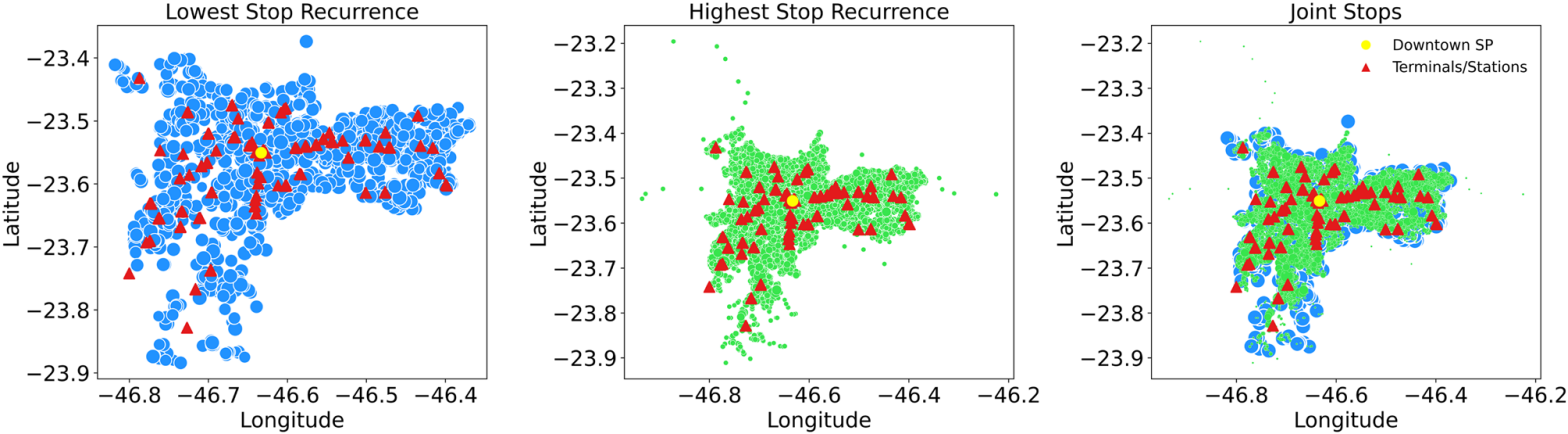
Geospatial Scatter Plot of Stops and Train/Metro Stations in São Paulo.

The distribution chart displayed in Fig. 5 provides a deeper analysis compared to the previous heatmap, focusing on trip frequency and the location of terminals and stations.

The first panel, Lower Frequency of Stops, identifies areas with fewer trips, marked by blue circles whose size reflects trip numbers. Smaller circles indicate less frequent stops and lower public transport density, while green triangles highlight terminals and stations as major connectivity hubs.

The second panel, Higher Frequency of Stops, showcases regions with more frequent stops, using green circles that grow with trip volume. These high-demand areas, such as São Paulo’s city center, benefit from dense and efficient transport infrastructure, ensuring better accessibility.

Finally, the third panel, titled Combined Stops, presents a combination of the previous analyses. It illustrates the overlap between areas of higher and lower frequency, emphasizing the centralization of public transport stops and their interconnections. The yellow dot in the chart marks São Paulo’s city center, a region of high public transport traffic and the main intersection point between various lines and modes. The visualization also highlights the unequal distribution of stops, showing that while the city center has excellent coverage, peripheral areas lack sufficient infrastructure and accessibility.

#### Geographical distribution of public transport stops

**Fig. 6:**
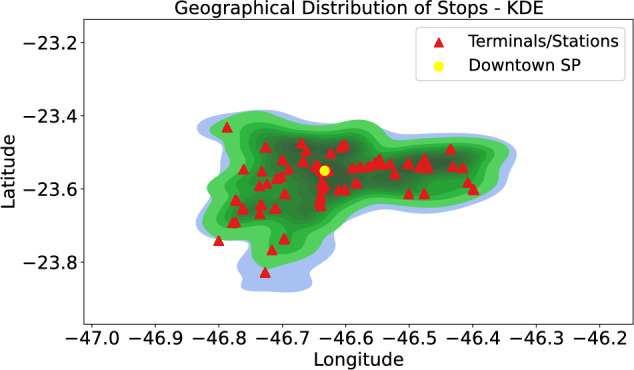
Geographical Distribution of Public Transport Stops with Train and Metro Points in São Paulo.

Following the same logic, the KDE (Kernel Density Estimation) visualization presented in Fig. 6 provides a smooth representation of the geographical distribution of public transport stops, enabling the identification of areas with higher activity concentrations. In this graph, zones with more intense colours indicate regions with higher stop density, while lighter areas reflect regions with lower concentrations.

Terminals and stations are represented by orange triangles, highlighting key transport hubs within the city. São Paulo’s city center, marked by a yellow dot, lies at the core of the dense distribution, confirming the higher concentration of stops in the central region. The graph also reveals that while the distribution extends to the peripheries, it does so with lower density, as observed in previous analyses.

This visualization is instrumental in understanding the spatial organization of São Paulo’s public transport system, analyzing coverage in central areas compared to the peripheries. It underscores potential inequalities in resource distribution and highlights the need for improved transport coverage in more remote regions.

### Predictive mobility performance

The first stage of the evaluation focused on assessing the predictive performance of three different regression models: *Decision Tree Regressor*, *Random Forest Regressor*, and *K-Nearest Neighbours Regressor*. All models were trained using a dataset of 208,794 valid records, derived from an initial pool of over 4.8 million raw entries. After preprocessing, 167,035 records were allocated for training and 41,759 for testing.

The Decision Tree model, after hyperparameter tuning, achieved a root mean squared error (RMSE) of 1047.57 seconds on the test set and a coefficient of determination ($R^2$) of 0.94. The Random Forest model, which builds upon the strengths of decision trees via ensemble learning, slightly outperformed the single-tree approach, obtaining an RMSE of 1038.83 seconds and the same $R^2$ score of 0.94. Although the performance difference in terms of accuracy was marginal, the Random Forest model required significantly more time for hyperparameter optimization (over 4179 seconds) compared to the Decision Tree (approximately 98.6 seconds), highlighting a trade-off between model robustness and computational cost.

The K-Nearest Neighbours (KNN) model, trained on standardized features, also performed well, achieving an $R^2$ score of 0.93 and an RMSE of 1162.28 seconds. While slightly less accurate than the tree-based models, the KNN algorithm benefited from a much faster optimization phase, completing in under a minute. Its performance suggests that local similarity-based predictions remain viable even in complex spatiotemporal transportation data, though they may fall short in capturing broader nonlinear patterns.

Among the three approaches, the *Random Forest Regressor* was selected as the final predictive model for scenario simulation, due to its slightly superior predictive accuracy and better generalization capabilities. This model served as the baseline for the subsequent evaluation of autonomous fleet scenarios, as described in the following subsections.

#### Autonomous vehicles on travel times

Following the selection of the optimized *Random Forest Regressor* as the primary predictive engine, two hypothetical scenarios were simulated to estimate the operational impact of integrating autonomous fleets into the public transport system. All results were derived from the dataset publicly available in the project’s GitHub repository (https://github.com/Lucas-HenriqueAntonio/SP_Public_Transport_Data_Analysis) . These scenarios were designed to explore how automation could enhance temporal efficiency by reducing segment travel durations under varying operational assumptions.

**Fig. 7:**
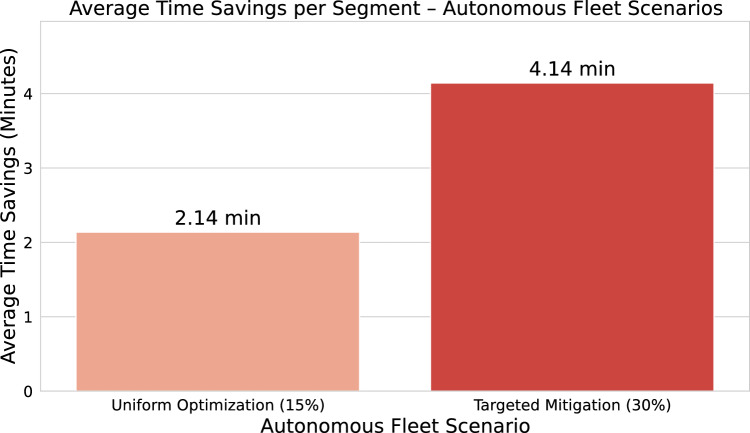
Estimated average time savings per travel segment across autonomous fleet optimization scenarios, based on Random Forest predictions.

Figure 7 provides a comparative summary of the simulation results, illustrating the average time savings (in minutes) achieved under the two proposed scenarios. The first scenario, *Uniform Optimization*, reflects a consistent system-wide improvement derived from uniform automation effects. In contrast, the second scenario, *Targeted Congestion Mitigation*, demonstrates a superior performance by concentrating optimization efforts on the most congested segments. The visualization highlights the pronounced advantage of the targeted strategy, particularly within heterogeneous urban environments where traffic conditions exhibit substantial variability.

The *Uniform Optimization*, applied a fixed 15% reduction to all predicted segment durations. This scenario models a broad deployment of autonomous vehicles, with general gains stemming from continuous route optimization and adaptive driving behaviour. The results demonstrated an average time saving of approximately 2.14 minutes per segment( Fig. 7). The *Targeted Congestion Mitigation*, introduced a more selective strategy: segments identified as slow (above the 75th percentile of predicted duration) were assigned a 30% reduction, while all other segments retained the standard 15% reduction. This approach simulates the capability of autonomous systems to dynamically reroute or overcome severe disruptions such as blockages or high-density traffic zones. The simulated average time saving in this case increased substantially to approximately 4.14 minutes per segment, nearly doubling the gain observed in the uniform scenario.

**Fig. 8:**
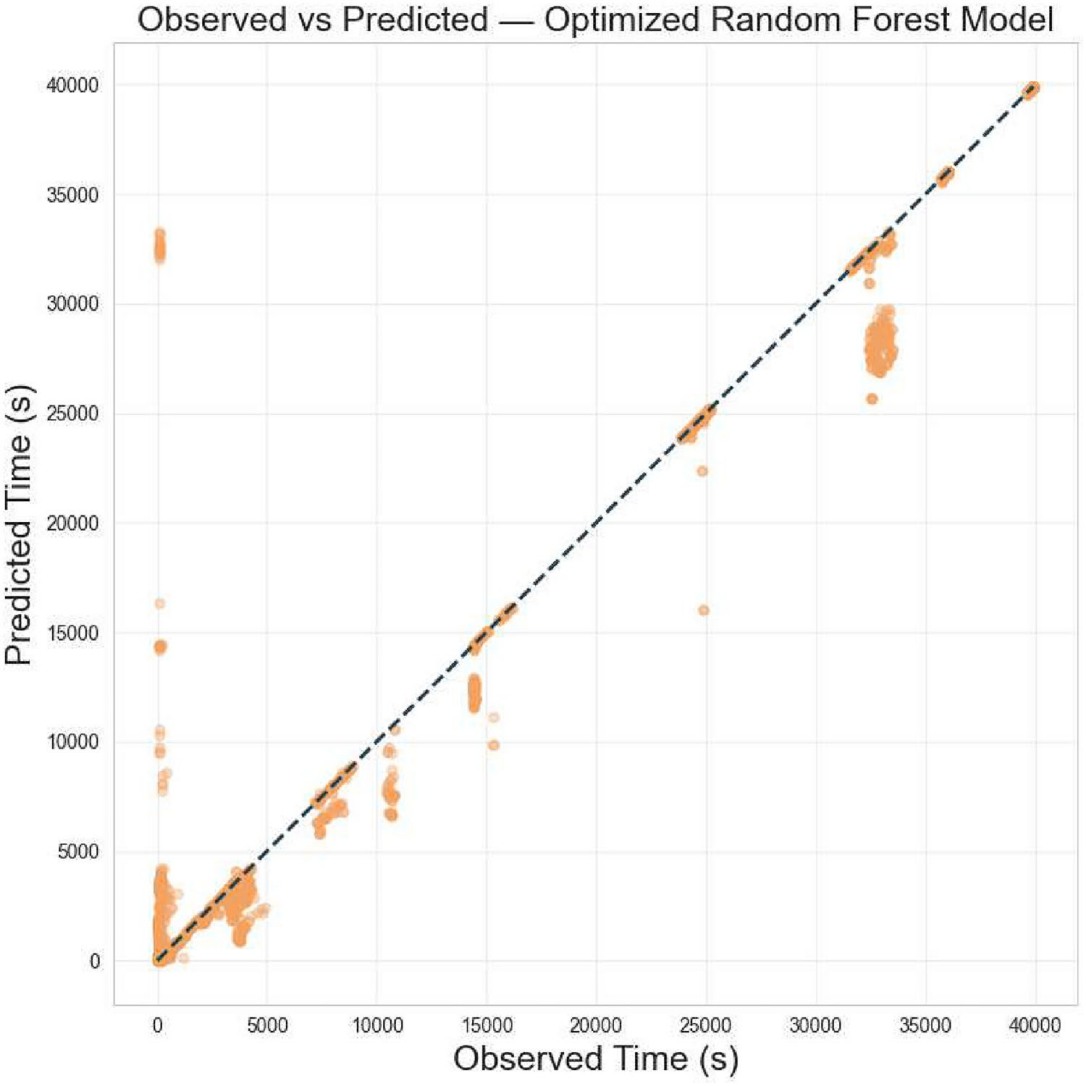
Observed versus predicted travel times from the optimized Random Forest model.

Figure 8 presents a comparative analysis between the observed and predicted travel times obtained from the optimized Random Forest regression model. Each point in the plot corresponds to an individual bus trip segment derived from the São Paulo GTFS dataset. The horizontal axis represents the empirically observed travel time, while the vertical axis displays the value predicted by the model. The blue diagonal line denotes the prediction reference, where the estimated and observed durations coincide. The overall distribution of data points is densely concentrated along this diagonal, indicating a high level of agreement between measured and simulated values. This visual alignment demonstrates that the model effectively learned the temporal dynamics underlying the operational behavior of the transit system, capturing the intrinsic variability of travel times across heterogeneous urban segments.

We can observe in Fig. 8a Minor deviations from the diagonal, most visible in longer travel segments, are consistent with the inherent stochastic nature of urban mobility, which is influenced by factors such as signal phasing, traffic congestion, passenger boarding variability, and route interference.

To evaluate the statistical reliability of the optimized **Random Forest** model, a *bootstrap* resampling procedure with 1, 000 iterations was performed to compute $95\%$ confidence intervals for the coefficient of determination ($\text {R}^2$) and the root mean square error ($\text {RMSE}$). The resulting estimates were highly consistent, with $\text {R}^2 = 0.943$ ($95\%$ $\text {CI} = [0.926$–0.958]) and $\text {RMSE} = 1038.83~\text {s}$ ($95\%$ $\text {CI} = [891.23$–$1176.91~\text {s}]$). The narrow confidence range and the fact that the $\text {R}^2$ interval lies entirely above zero confirm that the model’s explanatory power is statistically significant ($p < 0.05$). Similarly, the limited amplitude of the $\text {RMSE}$ interval indicates low sampling variability and stable predictive performance.

**Fig. 9:**
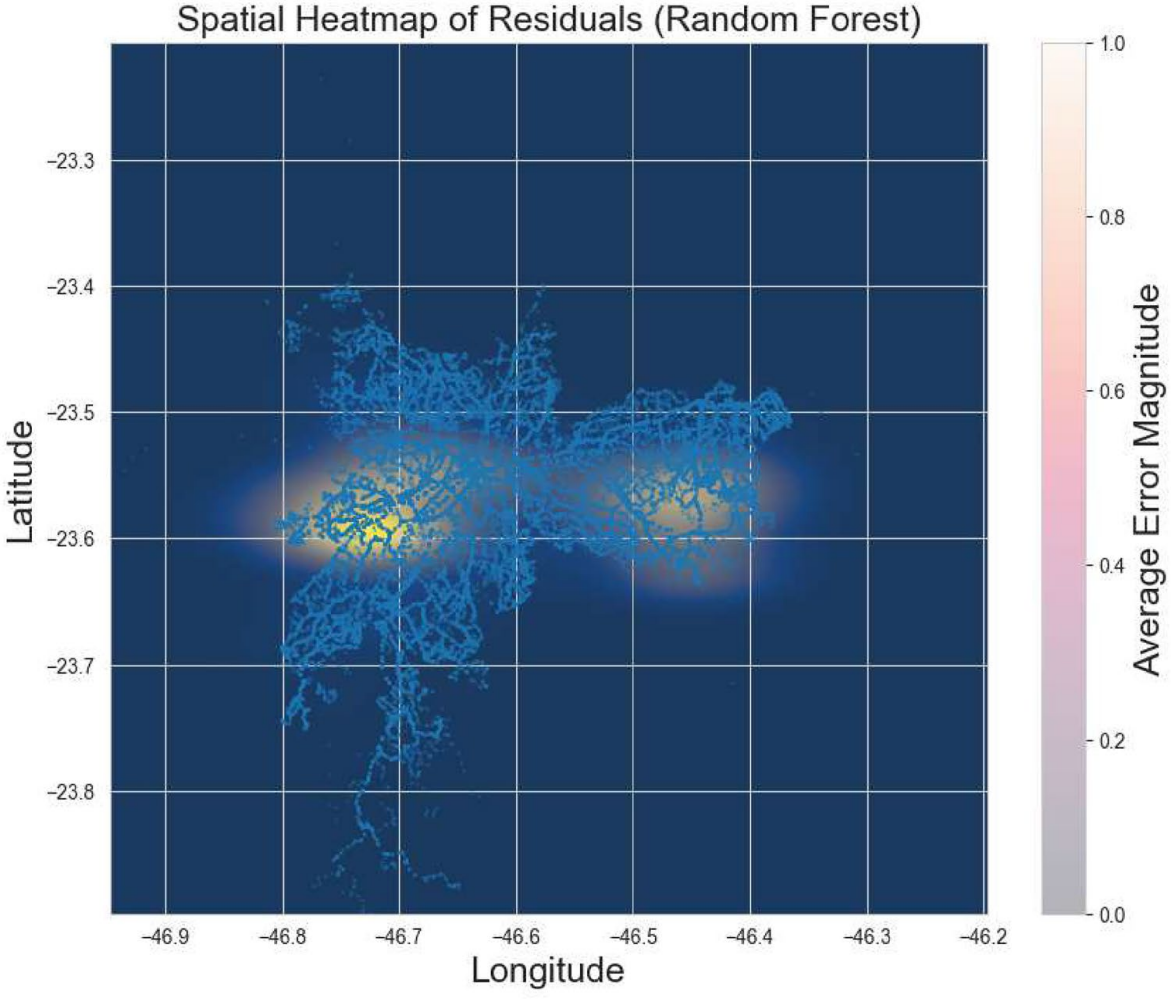
Spatial distribution of prediction residuals for the optimized Random Forest model. Lighter areas denote higher errors, highlighting regions where urban congestion reduces model accuracy.

Figure 9 presents the spatial heatmap of residuals derived from the optimized Random Forest regression model. This visualization integrates geographical information with model performance metrics to evaluate spatial consistency in predictive accuracy across São Paulo’s public transport network. The background color gradient represents the mean absolute error associated with each geographical segment: lighter tones indicate higher residual magnitudes (regions where the model tends to deviate more from observed values), while darker tones correspond to areas of lower error and higher predictive reliability. The overlaid blue trajectories depict the bus network routes analyzed in this study, providing a direct spatial reference between model accuracy and network topology.

The identification of localized regions where the model’s predictive capacity varies in response to complex operational dynamics. Higher residual concentrations are observed in the central and eastern sectors of the city areas characterized by heavy congestion, high stop density, and irregular signal coordination–conditions that introduce stochastic variability not fully captured by static GTFS data. Conversely, peripheral corridors and less congested routes exhibit darker regions, indicating greater model stability and precision. This pattern confirms that the Random Forest model effectively generalizes spatially, capturing most structural travel-time behaviors while revealing specific subzones where future enhancements could incorporate additional features such as dynamic congestion data or real-time signal timings.

#### Comparative analysis of predicted travel durations

To further extend the evaluation of temporal efficiency, a comparative analysis was conducted based on the absolute average predicted durations across all simulated scenarios. This assessment quantifies the overall magnitude of travel time reduction achieved through the integration of autonomous fleets, providing a direct measure of how each optimization strategy reshapes the temporal dynamics of the transit system.

**Fig. 10:**
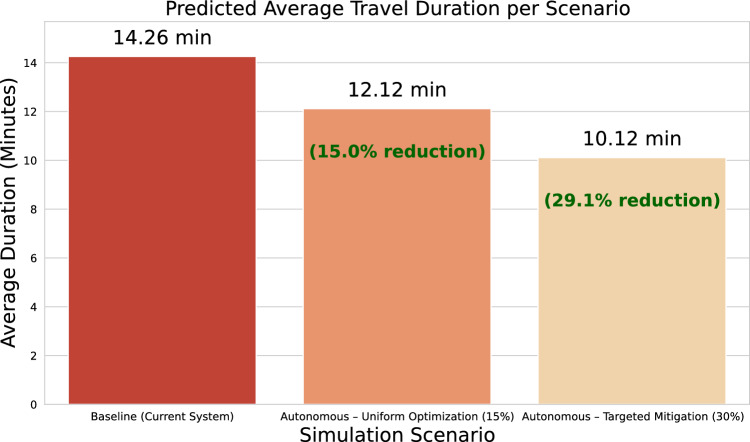
Predicted average segment durations under baseline and autonomous fleet scenarios, highlighting relative efficiency gains.

Figure 10 provides a comprehensive visual summary of these findings, comparing the predicted average durations across all scenarios. The figure clearly highlights both the absolute values and their corresponding percentage reductions, emphasizing the substantial performance gains achieved under the targeted strategy. The observed stepped improvement pattern reinforces the notion that adaptive optimization approaches–particularly those capable of identifying and prioritizing delay-prone segments–can deliver significantly higher operational benefits in complex and heterogeneous urban networks compared to uniform interventions.

From a simulation modeling perspective, our analytical approach differs substantially from most studies on AV integration, which predominantly employ agent-based or hybrid frameworks^13–15^. These models typically represent individual vehicle and passenger interactions to explore emergent mobility behaviors in synthetic urban environments. While such approaches provide rich behavioral insights, they depend on complex parameter calibration and are often limited in scalability and reproducibility for large metropolitan systems. In contrast, our study introduces a data-driven regression and simulation framework grounded in empirical GTFS operational data, which allows for direct, quantitative, and reproducible assessment of AV and VaaS integration at city scale. Rather than modeling agent interactions, our predictive modeling captures observed spatiotemporal dynamics through machine learning regression, offering a scalable method to evaluate automation impacts. This distinction positions our work as a complementary and empirically validated alternative to traditional simulation-based methods, bridging data-centric analytics and AV scenario assessment.

Therefore, in the baseline configuration, the average predicted duration for a travel segment was 14.26 minutes, representing the current system performance without any autonomous intervention. Under the *Uniform Optimization* scenario, applying a consistent 15% reduction across all segments, the mean predicted duration decreased to 12.12 minutes–corresponding to a 15.0% reduction relative to the baseline. In contrast, the *Targeted Congestion Mitigation* scenario, which applied a 30% reduction to slower segments and 15% to the remainder, further reduced the mean duration to 10.12 minutes, representing an overall 29.1% decrease.

### Autonomous vehicles and service expansion

A theoretical exploration was conducted on how autonomous vehicles could help improve coverage in underserved areas. This study now adapts those perspectives to the context of São Paulo’s public transport, considering the city’s specific challenges and exploring how this technology could benefit its peripheral and less-connected regions.

The integration of autonomous vehicles into São Paulo’s public transport system could improve accessibility in underserved peripheral areas, addressing challenges like unequal bus stop distribution and limited infrastructure through enhanced flexibility and efficiency.

In the context of São Paulo, the integration of autonomous vehicles into the public transportation system could significantly contribute to expanding coverage in areas with fewer stops and low vehicle frequency. Operating under an adaptive model, these vehicles would be able to adjust their routes and frequencies according to the specific demand of each area, efficiently meeting local needs. Areas such as the surroundings of Terminal Pirituba, which serve neighbourhoods like Perus and Jardim Paulistano, often face long waiting times and lower connectivity to other parts of the city. The introduction of smaller autonomous vehicles dedicated to connecting these neighbourhoods to the terminal could improve modal integration and reduce the isolation of these areas. Additionally, the flexibility in route creation would address temporary demands, such as events, fairs, or emerging needs from expanding communities. This adaptability would be particularly relevant for a dynamic city like São Paulo, where population growth and changing transportation needs require more scalable and connected solutions, complementing existing fixed routes ^41–45^.

The operation of autonomous vehicles in the Vehicle as a Service (VaaS) model would also bring benefits to São Paulo by improving traffic flow and redistributing transportation demand. In peripheral areas with congested roads during peak hours, autonomous vehicles could automatically adjust their routes in real time, collaborating with intelligent traffic control systems such as traffic light synchronization and traffic monitoring. This would not only alleviate congestion but also increase the predictability and regularity of transportation in these areas ^17,22,23,27,31^.

Furthermore, the reduction in operational costs, one of the most evident advantages of autonomous vehicles, could represent a relevant solution for São Paulo. In low-demand areas, the operation of conventional buses may face financial challenges, such as costs related to drivers and fuel. The transition to electric autonomous vehicles could mitigate these costs, making the public transportation system more economically viable in regions further from the center, where service may be limited. This way, it would be possible to sustain the expansion of public transportation more efficiently and without compromising the municipal budget ^20,37–39,41^.

Despite the transformative potential of integrating autonomous vehicles, their implementation faces significant challenges. The need for appropriate urban infrastructure, such as efficient communication networks and electric charging stations, is one of the main obstacles. Additionally, the creation of specific regulations and safety policies will be essential to ensure the safe and standardized operation of this technology. Another important challenge lies in public acceptance, as trust in automation and new mobility models is still developing ^4,5^.

Additionally, the VaaS (Vehicle-as-a-Service) model presents promising opportunities but requires the development of effective economic mechanisms, such as compensation and payment models, to encourage the participation of vehicle owners as part of the urban infrastructure. Overcoming these challenges will be crucial to consolidate a more efficient, sustainable, and inclusive public transportation system in São Paulo ^46,47^.

## Conclusion

The analyses presented in this study provided an understanding of the coverage and efficiency of public transport in São Paulo. Using geospatial visualizations such as heat maps, scatter plots, and Kernel Density Estimation (KDE) analyses, areas with low bus stop availability and long waiting times were identified, highlighting regions that require improvements to optimize urban mobility.

The integration of databases, using clustering, filtering, and spatial segmentation operations, enabled the creation of detailed and intuitive visualizations. These approaches highlighted significant disparities between central areas, characterized by higher bus stop density and connectivity, and peripheral neighbourhoods, which face lower vehicle frequency and less integration with public transport terminals.

Furthermore, the study proposed innovative technological solutions, such as the introduction of autonomous vehicles to expand coverage and improve connectivity in underserved areas. Electric autonomous vehicles, designed to operate economically and at scale, have the potential to offer more flexible and personalized services, such as routes adapted to local demands and greater integration with existing transport terminals.

The implementation of autonomous vehicles could reduce travel times and improve service frequency by replacing traditional vehicles. This would not only minimize road disruptions and congestion but also optimize traffic flow. In São Paulo, where such events severely impact mobility, adopting an autonomous fleet offers a crucial opportunity to increase public transport efficiency and reduce travel times.

However, the adoption of this technology still faces significant challenges. The implementation of autonomous vehicles requires a more robust urban infrastructure, including advanced communication networks and strategically distributed electric charging stations. Additionally, specific regulations to ensure safety and standardization still need to be developed, while public acceptance and trust in fully automated systems remain under development.

On the other hand, the Vehicle-as-a-Service (VaaS) model presents promising opportunities by transforming autonomous vehicles into integrated elements of urban infrastructure. This approach offers the possibility of creating a more connected and efficient mobility network, but its consolidation depends on viable economic mechanisms that encourage the participation of various stakeholders, such as vehicle owners.

In conclusion, the integration of advanced analytical tools and emerging technologies is essential for a more efficient and sustainable public transport system. However, its success depends on overcoming structural and economic challenges, enabling strategic interventions, especially in areas that most require improvements in coverage and service regularity. This will allow for more effective service to the population dependent on public transport.

The data that support the findings of this study are available in a GitHub repository (https://github.com/Lucas-HenriqueAntonio/SP_Public_Transport_Data_Analysis).

## Data Availability

The data that support the findings of this study are available in a GitHub repository https://github.com/Lucas-HenriqueAntonio/SP_Public_Transport_Data_Analysis.
